# Assessment of Burden of Malaria in Gwanda District, Zimbabwe, Using the Disability Adjusted Life Years

**DOI:** 10.3390/ijerph13020244

**Published:** 2016-02-22

**Authors:** Resign Gunda, Moses John Chimbari, Samson Mukaratirwa

**Affiliations:** 1 School of Nursing and Public Health, College of Health Sciences, University of KwaZulu-Natal, Howard Campus, Durban 4001, South Africa; Chimbari@ukzn.ac.za; 2 School of Life Sciences, University of KwaZulu-Natal, Westville Campus, Durban 4000, South Africa; Mukaratirwa@ukzn.ac.za

**Keywords:** disability adjusted life year, malaria, disease burden, Zimbabwe, Gwanda District

## Abstract

Malaria is one of the highest contributors to morbidity and mortality in Zimbabwe. However, there is paucity of knowledge regarding disability adjusted life years (DALYs) as a measure of burden of malaria in affected communities. The DALYs metric was used to assess the burden of malaria in Gwanda District with the aim of contributing to a better understanding of the impact of disease on affected communities. Data was collected from health facility malaria registers and the District Health Information System (DHIS) to estimate DALYs at household and district levels respectively. The household DALYs included 130 malaria cases from 2013 to 2015 while the DALYs for the district included 719 confirmed malaria cases from 2011 to 2015. Households lost a total of 153.89 DALYs with the majority of the disease burden (65.55%) occurring in the most economically productive age group (15–45 years) with a mean loss of 1.18 DALYs per malaria case. At district level, 251.09 DALYs were lost due to malaria and the calculated average district DALY rate for 2011–2015 was 36.29 DALYs/100,000 persons per year. It is important to estimate malaria burden to assist policy makers in making informed decisions when channelling resources for control and prevention of the disease.

## 1. Introduction

Malaria remains a challenge to public health in sub-Saharan Africa and is a leading cause of morbidity and mortality [1,2,3]. It is one of the most prevalent parasitic diseases worldwide, with thousands of people suffering every year [4]. According to the WHO World Malaria Report [5], a total of 104 countries are considered malaria endemic and at least 3.2 billion people are at risk. The same report states that a total of 198 million cases occurred globally in 2013 with most deaths occurred in Africa in children under the age of 5 years. Malaria contributes to a large public health burden with over 75% of the clinical episodes worldwide found in Africa [6]. In sub-Saharan Africa alone, mortality and morbidity due to malaria accounts for a loss of 35.4 million Disability Adjusted Life Years (DALYs) [7,8]. 

The Disability-Adjusted Life Year (DALY) is a metric measure for burden of disease [9] developed by the World Health Organization (WHO), World Bank and the Harvard School of Public Health researchers [10,11,12]. The 2010 Global Burden of Disease (GBD) study showed malaria as the seventh leading cause of DALYs lost globally [13]. The DALY not only looks at premature death due to disease, but also takes into account disability caused by disease. It combines both time lost due to premature mortality and non-fatal conditions. In the DALY approach, a disability weighted zero indicates perfect health (no disability), and one weighted 1 indicates death [11]. The DALY is a time-based measure that combines years of life lost due to premature mortality and years of life lost due to time lived in health states less than ideal health [12]. One DALY is defined as one lost year of “healthy” life, and the burden of disease (BoD) is a measurement of the gap between current health status and an ideal situation where everyone lives into old age, free of disease and disability [11]. Use of the DALY metric in assessing disease burden is essential as it is a measure used by most organisations to determine funding for malaria control in Africa [14,15]. Although the DALY is a measure of disease burden, it is also commonly used in the analysis of cost-effectiveness of interventions [16]. It therefore helps to indicate the socio-economic impact of disease burden unlike the conventional methods which only show the prevalence and mortality rates of the disease.

About 50% of the population in Zimbabwe lives in malarious areas therefore, nearly 6 million people are at risk of malaria [5,17]. The country experiences seasonal malaria transmission which is potentially epidemic [18]. Although there are on-going malaria control efforts in the country, the disease still remains a public health challenge [19,20] and continues to be a major cause of mortality and morbidity [21]. Resistance of the malaria vectors to insecticides [19,22] as well as resistance to anti-malarial drugs [23] contribute to the challenges in control of malaria in Zimbabwe. 

In addition to receiving government funding, the national malaria control programme in Zimbabwe also receives support from the Roll Back Malaria Partnership, the Global Fund to Fight AIDS, Tuberculosis and Malaria, and The President’s Malaria Initiative [5,24,25]. The Roll Back Malaria Programme was launched in Zimbabwe in 2001 and was subsequently scaled up [21]. The Global Fund to Fight AIDS, Tuberculosis and Malaria, and The President’s Malaria Initiative were launched in 2008 and 2009 respectively [24]. The recommended first-line treatment regimen changed from chloroquine or a combination of chloroquine plus sulphadoxine/pyrimethamine to artemisinin-based combination therapy, and the latter adopted by all health clinics by 2010 [21]. Diagnostic capacity improved, with rapid diagnostic tests (RDTs) available in health clinics by 2008. Mosquito control consists of indoor residual spraying and use of long-lasting insecticidal nets (LLINs) [21]. 

In order to assess the effectiveness of the current interventions to reduce malaria, there is need for in-country estimates of the national burden for the disease. This enables quantification of the needs for interventions to control and prevent malaria transmission such as indoor residual spraying and use of insecticide-treated bed nets in the population at risk. Estimates of the burden of disease also guide national disease surveillance strategies [26] and provide information on disease severity [26]. This assists in resource allocation as well as in targeting high priority areas. Most African countries have health budgetary constraints due to many competing health challenges and health system that are weak or dysfunctional [27]. It is therefore crucial that policy makers are provided with information on the economic benefits of malaria control and prevention. 

Limitations of the available data sources in many countries have led either to disease burden not being estimated at all or estimated using inaccurate methods [26]. It is important that calculations for disease burden are done at both the population and individual levels. A person may carry an important individual burden of disease, even though the population burden may be negligible [28]. Likewise, certain sub-populations may suffer a higher burden of disease than the average population burden. Previous studies on burden of disease in Zimbabwe focused on all-cause burden at national level [13,29]. The present study is the first to measure burden of malaria in Zimbabwe in terms of DALYs at household and district levels using locally-derived epidemiological data. This study was carried out as part of a larger study on malaria and schistosomiasis in Gwanda district, Zimbabwe.

## 2. Methods

### 2.1. Study Area and Population

The study was conducted in Gwanda District, the capital of Matebeleland South Province located south east of Zimbabwe. Gwanda is resident to 136,005 people (both urban and rural) of the 683,893 inhabitants of Matabeleland South Province [30]. There are 5656 households in the urban area compared to 26,773 in the rural area. Rural Gwanda is constituted of 24 wards which are administrative boundaries in the district and each ward is represented by an elected councillor. The low rainfall (<500 mm) received in the area has necessitated the setting up of irrigation schemes of various sizes in Gwanda district. People in the District mainly survive through subsistence farming, cattle ranching, brick moulding, irrigation farming, gold panning, fishing, vending and cross border trading. 

### 2.2. Data Collection

The main sources of routine malaria data in Zimbabwe are the National Health Information System (NHIS) and the Weekly Disease Surveillance System (WDSS). The NHIS receives information from the District Health Information System (DHIS). The DHIS forms the foundation for the country’s Health Management Information System (HMIS). Health facilities reporting to WDSS submit data to the districts which then transmit to provincial and central levels. Weekly meetings are held at the national level to review and discuss data quality, potential outbreaks, and action steps. Monthly reports on malaria cases and deaths from all public health facilities and mission clinics are reported through the DHIS. The malaria data captured include the number of suspected cases, the number of suspected cases with parasitological testing as well as number of confirmed cases. The current procedure is that all malaria cases are confirmed by rapid diagnostic tests (RDTs) which are available at all primary health care facilities. For every positive case, a blood slide is also prepared and sent to the district laboratory for further confirmation through microscopy. The DHIS information is reported via paper, from health facilities to district health information officers who enter these data into the DHIS electronic database. Consolidated electronic data are then reported to the relevant provincial office where data are consolidated and reported to the national level.

DALYs were determined at individual level as well as at district level. To calculate DALYs at the individual level, data was collected from a household census of all malaria cases that had been obtained from the health facilities in five wards in Gwanda South. The wards where data was collected were Buvuma, Nhwali, Ntalale, Selonga and Sengezane. The wards were purposively selected as they had the highest malaria cases in the district. Gwanda district has a total of 30 health facilities and each ward is serviced by one health facility (rural health centre or clinic). Cases were selected from the 2013/2014 and 2014/2015 malaria seasons and were obtained from malaria registers from 5 health facilities; 1 from each ward. Once selected, an interviewer administered household questionnaire was administered to the household head or their proxy. A household was defined as a person with his/her spouse, children and related or unrelated persons, who live together and constitute one unit [31] with a combined income stream. Only respondents above the age of 18 were eligible for inclusion. The household questionnaire was closed-ended and included questions on patient demographic details such as age, sex, date of sickness, duration of sickness as well as health seeking behaviour. Data for determination of DALYs for malaria at district level was collected from the District Health Information System (DHIS). Only confirmed malaria cases were included. Information collected included the total malaria cases, deaths, sex of patient, age of patient and incidences for malaria over a 5 year period from 2011 to 2015. All deaths were confirmed by a qualified medical professional and were from patients who were confirmed to have been malaria positive through both an RDT and a blood slide. This information was stratified by age and sex. 

### 2.3. Disability Adjusted Life Years Calculation

DALYs were calculated based on the standard method used by the 2010 Global Burden of Disease Study [13]. We slightly modified the method by using disability weights and life expectancies at death from the WHO life tables [32] instead of those from 2010 GBD Study [13]. The DALY for a population is calculated by adding the years of life lost due to premature death (YLL) and the years lost due to disability (YLD) giving the basic formula in Equations (1) and (2) [33,34,35]. We calculated the DALY burden at household and district levels using these formulae. No age weighting and discounting was applied.

DALY calculation at individual level;

(1) DALY=YLL+YLD

YLL = Life expectancy minus age of death

YLD = DW × L
DW = disability weight
L = duration of the case until remission or death

DALYs at individual level were calculated for each individual based on YLD and YLL for 130 malaria patients. The total number of DALYs was calculated by adding the DALYs for each individual. All cases of malaria that did not result in death were assigned YLL of 0. DALYs at individual level were calculated as the combined total of all individual DALYs lost from the study population due to malaria. Duration of a malaria episode was taken as 7 days for all the calculations. This was the calculated mean number of days for a malaria episode for our study sample (range 13 days; median 7 days; mode 7 days).

DALY calculation at district level

(2) DALY=YLL+YLD

where YLL = N × L

N = number of deaths
L = Zimbabwean life expectancy at age of death in years

where YLD = I × DW × L

I = number of incident cases
DW = disability weight
L = average duration of case until remission or death (years)

Previously, additional social preferences (discounting and age weighting) were considered when calculating DALYs in the 2006 Global Burden of Disease (GBD) study [10]. Discounting means that future gains and losses are counted less than if they had occurred today [11]. The years lost in the future were discounted, so that years lost now are worth more than years lost in the future. The 2006 GBD Study used a discount rate of 3% per year. Age weighting previously used in the DALY calculations was obtained from a scale where the value of a year lost rises steeply from zero at birth to a maximum at 25 years of age, and then decreases progressively in older ages. Because of weighting the value of the lifetime, the years of life in childhood and old age were counted less because social roles vary with age. The reason was that the young and the elderly often depend on the rest of society for physical, emotional and financial support [36,37]. However, due to controversies regarding the use of these social preferences, the 2010 GBD Study [13] did not use age weighting and discounting. Consistent with the 2010 GBD study we did not apply any social preferences.

Disability weights and life expectancy data for Zimbabwe was obtained from the WHO life tables [32]. A life expectancy of 63.5 for females and 58.9 years for males were used. We used average disease duration of 7 days for each malaria episode. This was the mean disease duration from the household survey.

In order to calculate YLDs, age specific disability weights were applied according to the DALY formula (Table 1). The disability weights range from 0 (perfect health) to 1 (death). Malaria data from the DHIS was not aggregated by all age groups but was only available as below 5 and above 5 age groups. This did not present a problem for the 0–4 year age group as the disability weight was the same in this group (Table 1). However for the greater than 5 year age group, a disability weight of 0.172 had to be applied as it represented the median age in that group. Disability weight for 0–4 was 0.211 while the disability weight for greater than 5 years age group was taken as that for median age of 40 years which is 0.172. All ages from 15 years and upwards had the same disability weight of 0.172.

### 2.4. Data Analysis

We calculated disease burden based on the methodology used in the Global Burden of Disease Study [13]. The parameters used for calculating DALYs as well as the sources of data are shown in Table 2.

## 3. Results

### 3.1. Malaria Burden at Household Level

Household level DALYs were estimated for 130 malaria cases from our study population in five selected wards. These were cases that were obtained from malaria registers from the health 5 health facilities in the wards. The 5 wards (Buvuma, Nhwali, Ntalale, Selonga and Sengezane) were purposively selected as they had the highest cumulative number of confirmed malaria cases from 2013 to 2015. At the district level, we estimated DALYs for a total of 719 reported malaria cases in the district from 2011 to 2015. 

DALYs at the household level were calculated from a total of 130 malaria cases (Table 3) from the five wards with the highest number of cases in the district. The number of malaria cases for males (60.8%) was higher than that for females (39.2%). The majority of cases led to remission with only 4% leading to death.

**Figure 1:**
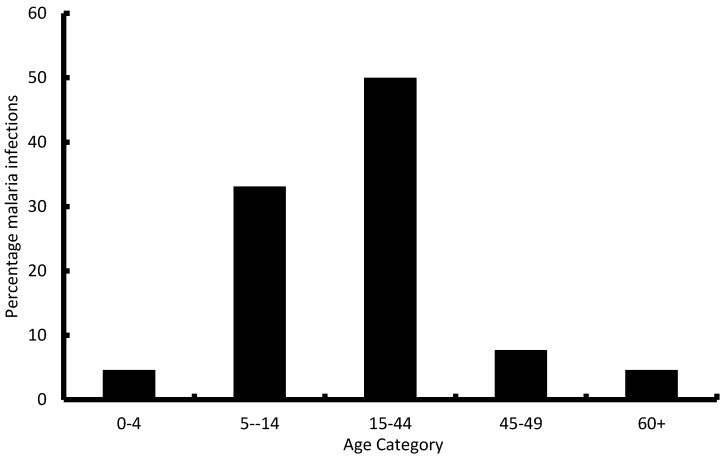
Age profile of malaria infected individuals from the household survey (*n* = 130) in 5 wards in Gwanda district of Zimbabwe.

The number of cases and deaths for the household level DALYs were grouped according to age groups as shown in Figure 1. The 15–44 years age category had the number of malaria cases (50.0%) followed by the 5–14 years age category (33.1%). The most economically productive age groups (15–60 years) had the majority of malaria cases.

At individual level, the total burden for 130 malaria cases from 2013–2015 in Selonga, Ntalale, Sengezane, Nhwali and Buvuma was 153.89 years of life lost (Table 4). The mean number of DALYs lost due to malaria per case was 1.18 DALYs. The majority of the disease burden (65.55%) occurred in the productive age category (15–45 years). YLLs constitute the majority of the burden of malaria compared to YLDs. This means that the malaria burden is largely due to mortality rather than morbidity in the study population. There were no malaria infections in the above 80 age category.

### 3.2. Malaria Burden at District Level

Data for 2015 shows malaria cases up to April 2015. The highest number of malaria cases were observed in year 2011 followed by 2014 (Table 5). A total of 719 confirmed malaria cases were reported in the district over the 2011 to 2015 period.

There were only 6 malaria deaths over the 2011 to 2015 period. Of these deaths, 5 were in 2011 while one was in 2014. No cases were reported in the other years.

As is the case with DALYs at the individual level, the majority of the malaria burden is from the YLLs rather than YLDs. There were a total of 251.09 DALYs lost due to malaria in Gwanda District for the period 2011 to 2015 and the majority of these (218.24 DALYs) were lost in 2011 (Table 6).

Table 7 shows the estimated yearly DALYs *per* 100,000 population *per* year. The calculated average district DALY rate for 2011–2015 for Gwanda was 36.29 DALYs/100,000 persons/year.

## 4. Discussion

Our study is the first in Zimbabwe to use local sources of information to assess burden of malaria at household and district levels. Most countries have estimated all-cause burden of disease at national level rather than disease-specific burden. One such study was carried in Zimbabwe [29]. Our study focused on malaria burden for the period 2011to 2015 because before 2011, reported cases were mostly based on clinical symptoms without confirmation through a rapid test or a blood slide. This meant that the number of reported malaria cases before 2011 in Zimbabwe were likely to be overstated. Hence malaria cases from 2011 onwards give a more accurate picture of the burden of malaria as all reported cases were confirmed. 

The results of this study show a general decline in the calculated number of DALYs lost due to malaria in Gwanda. This decline may be explained by the intensification of the malaria control intervention programmes in the district including case treatment, distribution of insecticide treated nets and indoor residual spraying (IRS). A total of 96% of cases reported led to remission of the diseases. Cases that led to deaths were mostly as a result of failure by the patient to present at a health facility before complications set in. It is also possible that some of the people who presented late at health facilities would have tried to seek treatment elsewhere. Alternative sources of treatment reported included traditional healers and faith-based healers. The high number of cases leading to remission shows the effectiveness of the current malaria treatment regimen.

Although the number of deaths from malaria was low, the few malaria deaths contributed to the bulk of the DALYs lost due to malaria through YLLs. A malaria episode lasted for 7 days on average. This short disease duration resulted in lower values for YLDs compared to YLLs. However, the burden of malaria in terms of YLDs would actually be higher if other possible consequences of malaria, such as renal complications, are taken into consideration [39]. 

Mortality was therefore a major contributor to DALYs for malaria. Malaria interventions that avert malaria deaths are therefore very critical as they significantly reduce the disease burden. 

Some previous studies on malaria burden reported higher burden in the below 5 years age category [39,40]. However, our results show a different scenario with most of the reported cases falling in the 5 years and above age group. This could be as a result of the many malaria interventions in the country targeting the below 5 years age group thereby resulting in very low number of cases in that group. This study showed that the majority of cases fall in the economically productive age groups (15–45 years) indicating that malaria has an economic impact on affected families as they lose productive time during sickness. There is therefore need for good investment in malaria control, not only to reduce the public health problem, but to also reduce the economic strain that malaria exerts on households.

DALYs are estimates and not definitive numbers but they indicate the relative size of the burden [41] and hence allow comparisons across different settings and geographical regions. Although mortality and morbidity due to malaria are showing a downward trend globally [5], it is important to have burden estimates for the areas that are still affected by the disease. The DALYs for the district showed variations between 2011 and 2015. If these DALYs were calculated over a longer period than the one in this study, they will have provided insight on the general trend of the burden of malaria in the area. This information will be important for policy makers as they plan for future control efforts. This shows the need for continued close monitoring of the burden of malaria as the variations from this study indicate that malaria control efforts are fragile. Outbreaks can easily occur if there are inconsistencies in the interventions for prevention of malaria. Since malaria is a focal disease, it is advisable for policy makers to continually implement geographically-targeted interventions for high burden areas as this may have a favourable cost-benefit ratio [42]. This will ensure that the disease burden is reduced and the possibilities of outbreaks are minimised. 

Other previous studies that utilised the DALYs in assessing burden of malaria and other neglected tropical diseases are shown in Table 8. The major difference with our study is that most of these studies applied age weighting and discounting in their DALY calculations [42,43,44,45,46,47,48,49] and the studies were done at national level [39,42,44,46,48,50]. 

Malaria is a notifiable disease in Zimbabwe and the majority of malaria episodes are captured in the DHIS. The DHIS is therefore a good representation of the malaria situation in the district. However, it is possible that some malaria cases may have not presented at the health facilities and hence not be included in the DHIS. Health facility-based data is also affected by many factors including accessibility, perceptions of care and whether or not self-treatment is available [52]. There is also a possibility of health workers not ascertaining malaria or failing to record cases even if they are diagnosed. Although the household census did not indicate any form of under-reporting by health workers it could still be a source of bias in the DHIS figures. We could not make direct comparisons between the household and district level DALYs because the data for the district was not disaggregated by age groups. There were only two broad groups of below 5 years and above 5 years. It was not logistically possible to collect age-specific data for the whole district during this study. Also, the data for the district did not classify deaths by sex whereas we were able to do in the household survey. This means that although our DALY estimates for the whole district gave us a good picture of the burden; the estimates from the household level data gave a better representation of the malaria burden. 

## 5. Conclusions

Developing countries such as Zimbabwe are faced with dwindling health budgets due to the prevailing harsh economic conditions. There is need for epidemiological data that is specific for local settings to provide guidance on how these limited resources can be allocated appropriately. This study provides data on malaria burden that can be used by policy makers in conducting targeted interventions to reduce the effects of the disease on rural poor communities. DALYs are useful in cost-effectiveness analysis of intervention programmes [53,54,55,56,57,58,59,60,61,62,63]. The results from the study can be used as baseline data for cost-effectiveness analysis of malaria intervention programmes in the district. Despite reported malaria cases in Zimbabwe showing a downward trend, it is still important for policy makers not to shift their attention from malaria interventions if the ultimate goal to eradicate malaria is to be reached. A lot of attention has been given to high transmission areas resulting in more resources being channeled towards these areas. This leaves the low transmission (pre-elimination) areas exposed to the effects of malaria burden especially at household levels as communities in these areas may not have adequate support. It would seem that assessing DALYs in low transmission areas is more critical as these areas are prone to resurgence and outbreaks if control efforts are relaxed due to the fewer number of malaria cases. However, it is important to use DALYs to assess burden of malaria in all endemic areas whether high or low transmission.

## Figures and Tables

**Table 1:** Disability weights for malaria for the different age groups (obtained from WHO life tables [38]).

Age Group (Years)	0–4	5–14	15–44	45–59	60+
Disability weights	0.211	0.195	0.172	0.172	0.172

**Table 2:** Parameters used for estimation of DALYs for malaria in Gwanda District of Zimbabwe.

Parameter	Data Required	Data Source	Comments
**YLL**	Number of deaths	Health facility records and health information system	Total deaths from 2013–2015 for individual DALYs and 2011–April 2015 for district level DALYs
	Zimbabwean life expectancy at age of death	WHO life tables for Zimbabwe (2013)	Obtained from the WHO website [32]
**YLD**	Disability weight	WHO Life Tables	Obtained from the WHO website [38]
	Duration of illness until remission or death (years)	Health facility records	Average duration of malaria until remission was 7 days (0.02 years)
	Number of incident cases	Health information system	

**Table 3:** Malaria cases at household level taken from a survey of 80 households in 5 wards in Gwanda district of Zimbabwe.

Sex	Total Malaria Cases (%)	Cases Leading to Remission (%)	Cases Leading to Death (%)
Males	79 (60.8)	76 (58.5)	3 (2.3)
Females	51 (39.2)	50 (38.5)	1 (0.8)
**Total**	**130 (100)**	**126 (97)**	**4 (3.1)**

**Table 4:** DALYs lost due to malaria (*n* = 130) in 5 wards in Gwanda district of Zimbabwe during the 2013–2015 malaria seasons.

Age (Years)	Total Reported Cases		Total Reported Deaths		Total YLLs	Total YLDs	Total DALYs
	Males	Females	Males	Females			
0–4	5	1	0	0	0	0.02	0.02
5–14	26	17	1	0	52.90	0.07	52.97
15–29	32	14	1	0	44.50	0.11	45.51
30–44	9	10	1	0	29.80	0.05	29.85
45–59	4	6	0	1	25.50	0.02	25.52
60–69	3	2	0	0	0	0.02	0.02
70–79	1	0	0	0	0	0.003	0.003
80+	0	0	0	0	0	0	0
Total	79	51	3	1	152.70	0.29	153.89

**Table 5:** Malaria confirmed cases from 2011–2015 for Gwanda district of Zimbabwe.

Year	Males, under 5 Years	Males, 5 Years+	Females, under 5 Years	Females, 5 Years+	Total Males	Total Females	Grand Total
2011	3	191	0	108	194	108	302
2012	3	51	0	19	54	19	73
2013	6	61	4	45	67	49	116
2014	0	93	7	69	93	76	169
2015	4	25	4	26	29	30	59
Totals	16	421	15	267	437	282	719

**Table 6:** Disability Adjusted Life Years (DALYs) lost due to malaria in 5 wards in Gwanda district, Zimbabwe, in 2011–2015.

Age (Years)	Year	Total Reported Cases		Total Reported Deaths	YLLs	YLDs	DALYs
****	2011						
****		Males	Females				
**<5**		3	0	2	217.2	1.04	218.24
**≥5**		191	108	3			
****	2012						
**<5**		3	0	0	0	0.25	0.25
**≥5**		51	19	0			
****	2013						
**<5**		6	4	0	0	0.41	0.41
**≥5**		61	45	0			
****	2014						
**<5**		0	7	0	31.6	0.59	32.19
**≥5**		93	69	1			
****	2015						
**<5**		4	4	0	0	0.21	0.21
**≥5**		25	26	0			
**Totals**		437	282	6	248.8	2.50	251.09

**Table 7:** Estimated burden of malaria at the population level in Gwanda District of Zimbabwe.

Year	Total Population	Total Reported Cases	Incidence Rate/1000 Population	Total DALYs	DALYs/100,000 Population/*per* Year
2011	142,202	302	2.12	218.24	153.47
2012	115,778	73	0.63	0.25	0.22
2013	116,936	116	0.99	0.41	0.35
2014	118,105	169	1.43	32.19	27.25
2015	119,286	59	0.49	0.21	0.18

**Table 8:** List of studies that have utilised the DALY metric for burden of assessment.

Ref.	Year	Disease	Study Coverage	Place/Country	Age Weighting	Discounting	Total DALYs
[39]	2007	Malaria	National	Sudan	No	No	2,877,000 in 2002 in Sudan
[43]	2004	Echinococcosis	District	Tibet	Yes	Yes	32,978 (0.81 DALYs lost *per* person)
[44]	2005	Dengue	National	Thailand	Yes	Yes	427 *per* million *per* year (year 2001)
[45]	2008	Human African Trypanosomiasis	District	Uganda	Yes ^a^	Yes	1157.32 ^b^
[42]	2014	Human African Trypanosomiasis	National	Uganda	Yes	Yes	486.3 *per* 100,000 persons *per* year
[50]	2009	Chikungunya	National	India	No	No	45.26 *per* million *per* year (year 2006)
[46]	2009	Dengue	National	Brazil	Yes	Yes	22 *per* million *per* year in Brazil
[47]	2010	Human African Trypanosomiasis	District	Tanzania	Yes	Yes	215.7 (from 143 HAT patients)
[48]	1998	Dengue	National	Puerto Rico	Yes	Yes	658 *per* million *per* year
[49]	2009	Cysticercosis	Provincial	West Cameroon	Yes	Yes	9.0 *per* thousand persons *per* year
[51]	2013	Dengue	Regional	South-east Asia	Not stated	Not stated	372 *per* million people *per* year

^a^ The study calculated DALYs with and without age weighting; ^b^ Assuming 69% under-reporting without age weighting.
